# Recent Trends in the Design, Synthesis and Biomedical Applications of Covalent Organic Frameworks

**DOI:** 10.3390/polym15010139

**Published:** 2022-12-28

**Authors:** Gagandeep Kaur, Dinesh Kumar, Subramanian Sundarrajan, Seeram Ramakrishna, Pawan Kumar

**Affiliations:** 1 Materials Application Research Laboratory (MARL), Department of Nano Sciences and Materials, Central University of Jammu, Rahya Suchani, Jammu 181143, India; 2 Department of Pharmaceutical Sciences, Central University of Haryana, Mahendergarh 123031, India; 3 NUS Centre for Nanotechnology and Sustainability, Department of Mechanical Engineering, Lower Kent Ridge, National University of Singapore, Singapore 117581, Singapore; 4 Department of Prosthodontics, Saveetha Dental College and Hospitals, Saveetha Institute of Medical & Technical Sciences, Saveetha University, Chennai 600077, India

**Keywords:** COF technology, synthesis, applications, biomedical utility

## Abstract

The most recent and advanced class of crystalline and permeable compounds are covalent organic frameworks (COFs). Due to their exceptional qualities, such as their porous structure, high surface area, strong chemical and thermal stabilities, low density, good water stability, luminescent nature, and so on, COFs have seen remarkable growth over the past ten years. COFs have been successfully researched for a number of applications based on these characteristics. The current state of COFs has been reported in this study, with particular attention paid to their design, topology, synthesis, and a variety of biological applications, including drug delivery systems, photodynamic and photothermal therapy, biosensing, bioimaging, etc. Moreover, several miscellaneous applications, such as catalysis, gas storage and separation, photocatalysis, sensors, solar cells, supercapacitors, and 3D printers, have also been explored. It is significant that we have examined current research on COFs with a focus on the biological applications, which are infrequently covered in the literature. Descriptions of the difficulties and prospective outcomes have also been given.

## 1. Introduction

The term “covalent organic frameworks” (COFs) refers to a newly discovered category of materials that have a chemically permeable organic structure. In principle, COFs are crystalline materials made of a variety of organic ligands that are covalently connected to create long-range extended structures. The production of crystals in COFs has been accomplished through various approaches that establish the optimal balance between the thermal reversibility of the linking processes and the dynamics of the corresponding crystals [1]. The main components that make up their spine are composed completely of light elements, including oxygen, silicon, hydrogen, carbon, and nitrogen, among others. As a result, COFs have evolved to comprise organic units joined by robust connections, such as -B-N, -B-O-Si-, -B-O-, -C-N-, and so on [2,3].

COFs can be classified either as two-dimensional (2D) or three-dimensional (3D) COFs, depending on the dimensions of the construction blocks. In 2D COFs, the framework of covalent bonds is restricted to 2D sheets. These sheets are then stacked on top of one another to generate a layered structure with overlapping layers that presents periodic columns. This columnar pileup structure offers a novel approach to the construction of an ordered solid network, which is traditionally difficult to achieve through the use of conventional covalent and noncovalent approaches. Since the ordered columns in 2D COFs are able to enhance charge carrier transport in the stacking direction, this indicates that 2D COFs have the potential to be used in various applications. In contrast, the framework of a 2D COF can be extended into the third dimension by using a building block that contains a sp^3^ silane or a carbon atom. Low densities, a variety of open sites, and high specific surface areas are all visible in the 3D COFs. These excellent properties encourage studies of 3D COFs for drug delivery applications [3,4].

**Figure 1:**
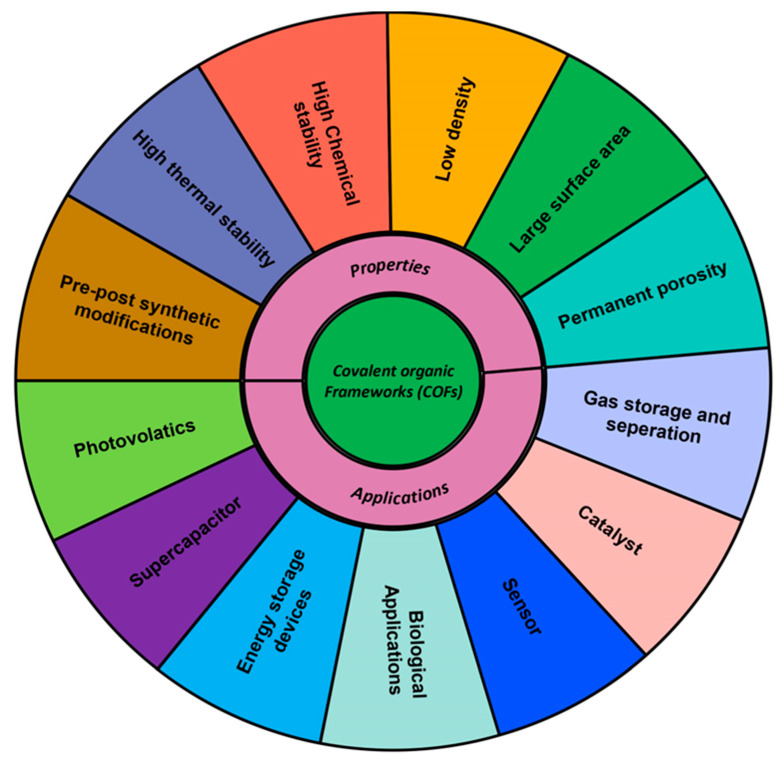
Properties and applications of COFs.

In addition to the dimension of COFs, they exhibit important features such as structural versatility, easy surface functionalization, pre- and post-synthetic modification, excellent chemical and thermal stabilities, a high surface area, permanent porosity, and low density [4]. The COF materials have significant advantages in a variety of applications thanks to their customized functions and well-defined crystalline porous architectures. Importantly, unlike MOFs, the majority of COFs have lower densities and, hence, exhibit good stability in chemical reagents, even under acidic, basic, oxidizing, and reducing conditions [3,4]. Additionally, COFs can also endure severe chemical and thermal conditions while maintaining their ordered structure and crystallinity. Commanding these features, COFs can be used for variety of applications which include energy and gas storage, optoelectronics, adsorption, drug delivery, Chemo sensors, and separation. Other intriguing applications include catalysis and sensing [5,6,7]. Figure 1 given below describes the properties and applications of COFs.

When compared to other nanosystems, COFs offer several benefits that make them preferable for use in nanomedical applications. These benefits are based on their excellent properties, such as structural periodicity, internal channels, high porosity, and pores (which can help to facilitate the easy transfer of medicinal substances, while at the same time allowing for substantial doses). Furthermore, the diversity of COFs can be easily incorporated by carefully choosing and functionalizing organic building blocks. Because of their versatility, COFs can carry and release medications or theranostic agents under precise control, thereby highlighting their special function as diagnostic and therapeutic nanoparticles in synergistic anticancer therapy. Additionally, COFs exhibit exceptional chemical stability and biocompatibility due to their strong covalent bond coupling and nonmetallic characteristics. Recent research has shown that COFs can be used as drug carriers, due to their excellent characteristics, such as high loading efficiency, exceptional release properties, and minimal systemic toxicity. Therefore, using COFs for tumor-targeted anticancer drug delivery in vivo will be quite appealing. Because of their high sensitivity, non-intrusiveness, quick response time, and real-time performance testing, they have also been used in fluorescence imaging to study the roles of biological molecules in a natural microenvironment. Utilizing COFs’ distinctive properties, including the long-range crystal state and eclipsed stacking assembly, they are applied as potential fluorescence imaging agents. Hence, in this part, we have emphasized the uses of COFs for fluorescence imaging [3,4,5,6,7,8].

**Figure 2:**
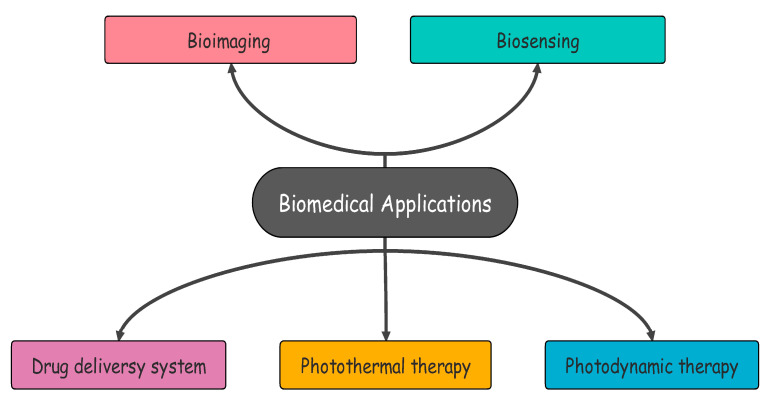
Biomedical applications of COFs.

Despite the significant amount of research that has been conducted over the last few years, the application of COFs in cancer treatment is still mostly unknown. Due to the inadequate development of COFs from the perspective of cancer therapies, research into the potential use of COFs in cancer therapy is lacking so far. Therefore, the utilization of COFs in nanomedicine requires additional research. To fully make use of the promising potential of COF-based nanosystems in cancer treatment, one needs to have a sufficient understanding of these systems [8,9]. As of now, research on COF-based nanosystems has been conducted on a variety of subjects, such as bioimaging, drug delivery, phototherapeutics, and antibacterial agents [9]. The main aim of this work is to explore biological applications of COFs, challenges and future perspectives in this field. Some of the biomedical applications of COFs are given in Figure 2.

**Figure 3:**
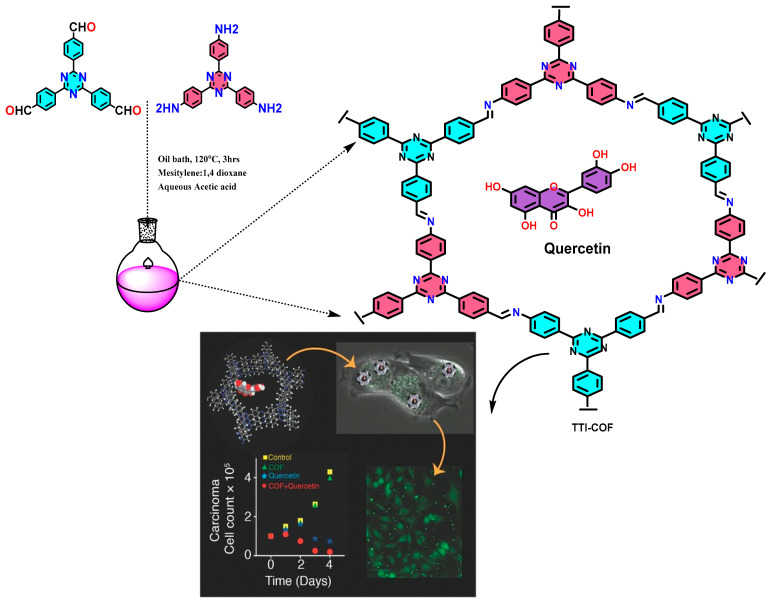
Graphical synthesis representation of imine-linked TTI-COF tagged with quercetin for cancer treatment [11].

To explore the biomedical applications of COF, covalently grafted porphyrinic PS (POR), VONc@COF-Por, upon visible (red LED) and NIR (808 nm laser) irradiation displayed high ^1^O_2_ generation. As reported, it exhibits a 55.9% photothermal conversion ability [10]. Additionally, this demonstrates an effective combination for photothermal and photodynamic therapeutic impact on Michigan Cancer Foundation–7 (MCF-7) tumor cells both in in vivo and in vitro tests [10]. In recently reported work, TTI-COF showed efficient biocompatibility, photothermal conversion (39.3%) and quantum yield (10.26%) of hydroxylating edge confined COF [11] (Figure 3).

Encapsulation of quercetin-loaded COFs with human breast carcinoma cells has essentially been performed to show the improved permeability and retention effect (EPR) in comparison to simple quercetin molecules. Furthermore, the increased rate of drug loading and prolonged release has also been found due to host–guest interaction and strong hydrogen bonding [11].

**Figure 4:**
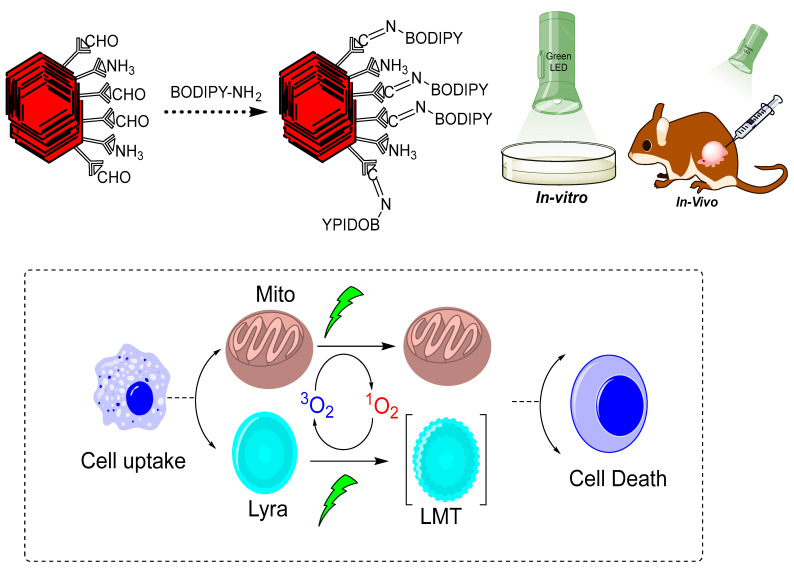
Graphical representation of LZU-1-BODIPY-2 for photodynamic therapy [12].

In another study, the synthesis of BODIPY-NCOF (boron-dipyrromethene nanoscale COFs) was reported via a Schiff base condensation reaction between the free aldehydic group of benzene-1,3,5-tricarbaldehyde and the imine group from tert-butyl(4-aminophenyl) carbamate (NBPDA) Figure 4 [12]. The resulting monoamine-functionalized BODIPY was bonded to the free aldehyde group on LZU-1 through a bonding defect functionalization approach for LZU-1-BODIPY-21. The lab-synthesized COF was examined on HeLa and Michigan Cancer Foundation-7 (MCF-7) tumor cells with a low-power red-light-emitting diode (LED) to investigate its photodynamic and photothermal properties. As an ^1^O_2_ detector, 1,3-diphenylisobenzofuran (DPBF) was used to assess the COF’s photodynamic activity. The absorbance of DPBF was lowered to 20.8% of its initial value after being subjected to a red LED (50 mW/cm^2^) for 7 min, indicating very efficient ^1^O_2_ production. When the PBS dispersion of the same COF was illuminated with an 808 nm laser of 1.5 W/cm^2^ intensity, no substantial decrease in DPBF absorbance (92.3%) was found, indicating that an NIR light source would not generate effective ^1^O_2_ production. There was no increase in ^1^O_2_ generation efficiency when both the red LED and the 808 nm laser were applied, compared to when only the red LED was used. As a result, the red LED was the source of ^1^O_2_ generation [12].

Likewise, we discuss more examples on the biomedical applications of COFs, with special emphasis on drug delivery, photodynamic and photothermal therapy, bioimaging, biosensing and biological analysis in Section 4. Furthermore, the design principle, chemistry, topology, and different synthesis methods of COFs are described in Section 2 and Section 3, respectively. Overall, we have discussed the recent investigations on COFs with special emphasis on biomedical applications. Apart from these various aspects, biological applications have been explored based on the available literature. Finally, we attempt to describe the challenges and future perspectives in this field, which will be useful to researchers already working in the field or those who will start working in this area in the future.

## 2. Chemistry, Design Principle and Topology of COFs

### 2.1. Chemistry of COFs (Reticular Chemistry and Dynamic Covalent Chemistry)

**Figure 5:**
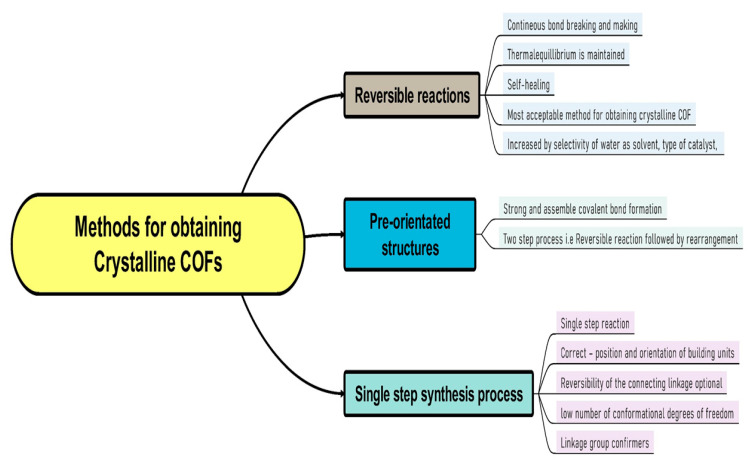
Three different methods to obtain good crystallinity COFs.

**Figure 6:**
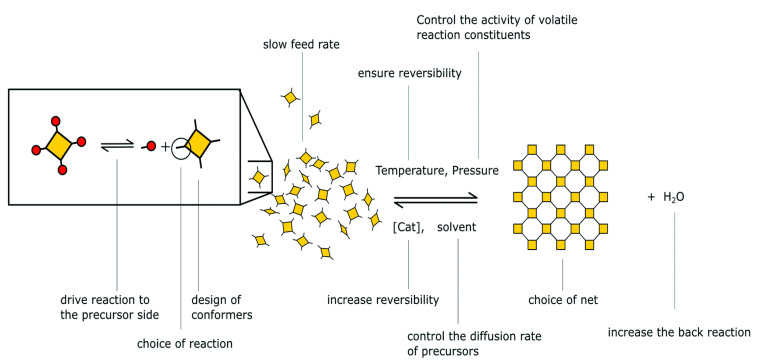
Complete schematic representation to obtain highly crystalline COFs [5].

The reticular chemistry involves strong bonding to join molecular basic components to form crystalline open structures. It has greatly extended the range of chemical compounds and materials such as organometallic compounds (OMC), metal organic frameworks (MOFs), covalent organic frameworks (COFs), and so on. The linking processes in COFs and MOFs had to be developed to solve the “crystallization challenge”, resulting in crystalline products whose structure could be determined unequivocally using X-ray and electron diffraction analysis tools [13]. The crystallinity of COFs has shown how the outer surfaces of their frameworks may be functionalized to create locations for post-synthesis covalent reactions via chemical conjugation [13]. Reticulation of molecular building blocks into structures to produce COFs can circumvent quenching induced through aggregation. It is the most common emission quenching mechanism. For example, a very luminescent COF with organic polygons as vertices resulted in a new aggregation-induced emit [14]. This type of architectural modification results in crystalline porous COFs with regular layered columns patterns that dominate the COF’s luminance. Due to a topological interlocking effect of interlayer chemical bonds and interlayer non-covalent interactions, it has attained a phenomenal quantum yield [14]. In recent years, different approaches for creating regular materials by relying on reversible processes have sparked a great deal of attention [12,13,14]. Highly potent methods to regulate covalently linked substances produce superior crystallinity and sustainability in COFs while tolerating chemical functionalities with reversible reactions. The methods for obtaining crystalline COFs may be divided into three categories, i.e., (A) based on reversible reactions, (B) based on pre-orientation of construction materials, and (C) based on a single step synthesis route [12,13,14,15,16]. Figure 5 and Figure 6 represent flow diagrams including all three categories to enhance the crystallinity of COFs and the complete strategy to increase the crystallinity of the synthesized COFs.

In recent years, the most popular approach to achieve crystalline COFs is to crystallize them via reversible covalent bond formation. Essentially, reversible reaction allows continual bond creation and breaking, and furthermore, defect correction during the construction of the building blocks. Therefore, the system can eventually reach the thermodynamic equilibrium conditions for the final product, whereas in pre-orienting the construction components, the initial or primary step is isolated from the development of strong covalent bonds [15]. Weak connections are employed to align the building blocks, allowing for simple crystallization and reversibility of the ordering process. In a subsequent phase, stronger bonds are used to link the building components. This technique includes everything from generating molecular crystals that are subsequently photochemically crosslinked to reversibly crystallizing COFs and then irreversibly converting the labile bond into a more stable connection. The conformational strategy of COFs and their constituent components is such that joining foundations can only take place in only one position and orientation, rendering reversibility of the bridging linkage acceptable [16]. Basic components are usually aligned through utilizing strong connectivity to other basic units, and order is accomplished via a small number of structural degrees of freedom [16]. This technique has also been used to improve the crystallinity of COFs through reversible reactions via decreasing the number of potential conformers [16].

### 2.2. Design Principle and Topology of COFs

The designing principle behind the COF is a condensation reaction. Polymerization processes are centered on the self-condensation of a substituted monomer or the co-condensation of monomer units with two distinct functional groups. In the self-condensation process, single monomers with active functional groups are combined, whereas in the co-condensation reaction, the reaction between distinct monomers with distinct or the same functional groups is combined. To produce a polymer skeleton with an extended 2D structure, covalently connecting monomers requires a planar or linear structure of subunits with symmetry. Essentially, the topologies are aligned to satisfy a topologically directed 2D polymer formation, in contrast to a knot with a 3D branching geometry, i.e., a tetrahedral geometry (T_d_) is required for 3D growth [17].

Co-condensation of ether two monomers or self-condensation of one monomer results in symmetric topology. Co-condensation joins two monomers as a knot and a connector to produce a polygon structure that is alternatively connected. A [C_3_ + C_2_] design, for instance, produces a hexagonal topology by combining C_3_- and C_2_-symmetric monomers, a [C_4_ + C_2_] design creates a new main topology tetragonal structure with C_4_ and C_2_ symmetric monomers, whereas rhombic topology is produced when C_2_ monomers undergo self-condensation to produce a [C_2_ + C_2_] design diagram [17]. The direct fabrication of microporous tetragonal skeletons is possible using a [C_4_ +C_4_] geometry diagrams, whereas the microporous hexagonal topology is framed by the self-combination of C_3_ monomeric units, resulting in a [C_3_ + C_3_] design.

Additionally, the Kagome honeycomb, with an octagonal mesopore in the center and six trigonal micropores on each corner, was formed by linking a C_3_-symmetric knot with a C_2_-symmetric linker framework. Furthermore, a tetrahedral (T_d_) or orthogonal (oh) knot in conjunction with C_1_, C_2_, C_3_, C_4_, and T_d_ organic linker types results in 3D COFs with connections in various combinations to create skeletons and pores [18]. COFs in three dimensions tend to fold into giant covalent structures. Additionally, the most recently utilized precursors are with active groups containing tetrahedral building blocks, which can condense with linear or triangular molecules via [4 + 2] or [4 + 3] condensation reactions that result in the formation of dia or ctn/bor topological networks, respectively. For the fundamental topological design, on the other hand, the methods for creating 3D COFs were confined to these two types for a long time, which severely limited the many significant functional units that were combined. Therefore, to build 3D COFs, new topology design methodologies are being developed [19,20]. In 2016, a revolutionary points topology design was created to address these enduring issues in the design and topology of 3D COFs. Additionally, four-sided functionalized molecules (such as pyrene and porphyrin) when inserted into tetrahedral (Td) secondary units, expanding their topological family in order to form 3D COFs via [4 + 4] condensation processes [21].

**Figure 7:**
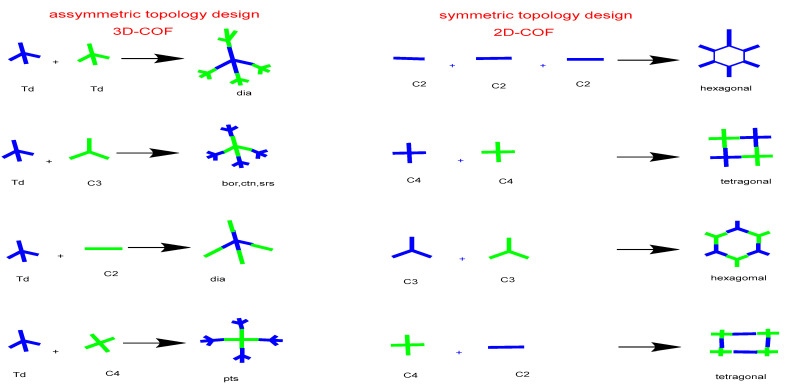
Topology diagram of symmetric and asymmetric linkers reported for COFs synthesis.

Apart from this, 3D COFs’ condensation type, topology type and monomer symmetry present in recently reported COFs in the literature are listed in Table 1 and Figure 7. Researchers can learn from these lists to design new COFs with specific properties for diverse applications.

## 3. Synthesis Method of COFs

**Figure 8:**
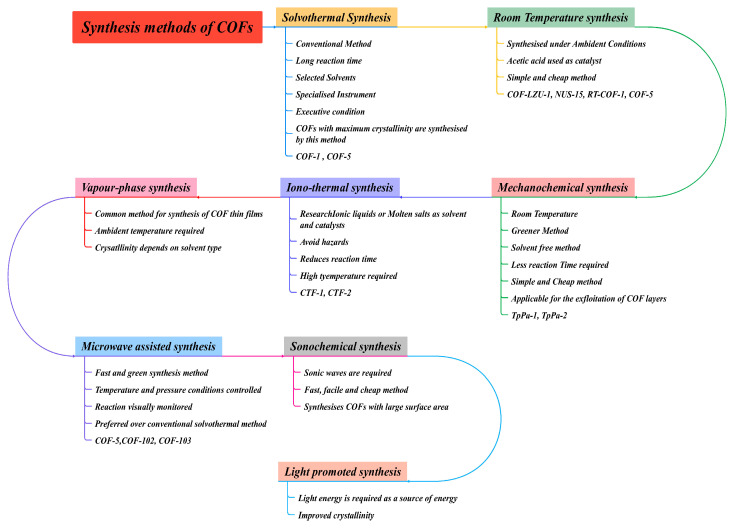
Different synthetic routes for COFs synthesis as per the available literature.

To date, a significant number of synthetic methods have been identified, such as solvothermal, ionothermal, mechanochemical, microwave-assisted, and ultrasonication processes, as given in Figure 8. It is essential to design acceptable circumstances for reversible bond formation under certain experimental conditions for COF synthesis. Importantly, the solvothermal technique is one of the most common synthesis methods for COFs synthesis with good crystallinity. In this section, we discuss different synthetic techniques of COFs as reported in the recent literature. Table 2 gives different COFs with its suitable conditions.

### 3.1. Solvothermal Synthesis

In the solvothermal synthesis technique, monomers and mixed solvent systems are inserted and degassing is performed by multiple freeze–pump–thaw cycles in a Pyrex tube. After that, the tube is sealed and subsequently heated to a specific temperature for a specific reaction time. COF solvothermal synthesis lasts 3–7 days and demands elevated heating in a sealed tank. In this method, the reaction system’s pressure is critical, since it can have a substantial impact on the COF microstructure and porosity. Likewise, choosing the reaction media and different factors, including the solubility, the rate of reaction, crystal formation, the ‘self-healing’ structure, and the crystal growth rate, is crucial for COF synthesis [22]. Furthermore, the solvent used for COF synthesis is important due to its control of the solubility of the reactants and is linked to reaction rate, crystal formation, crystal overall growth, and error correcting structures. As a result, determining the best solvent solution for synthesizing crystalline COFs has proven to be a challenging task. In the presence of an acidic catalyst, COFs are typically produced using a binary solvent combination: the precipitate is gathered, washed with the necessary solvents, and then subsequently dried under vacuum to obtain COFs as a solid powder.

For example, boronate ester-linked COFs have been synthesized using solvent combinations including dioxane–mesitylene, THF–methanol, and DMAc–o-dichlorobenzene [23]. The first 2D COF, i.e., COF-1, was built as a multilayer hexagonal structure using a self-molecular dehydration process of 1,4-benzenediboronic acid (BDBA), whereas COF-5 synthesis was reported with a coaxial stretched sheet structure using a condensation reaction of BDBA and 2,3,6,7,10,11-hexahydroxytriphenylene (HHTP). Furthermore, a sealed environment ensures the convenience of media for effective crystalline development of the COF [10]. Nevertheless, a long reaction time, i.e., 3–7 days, and the requirement of elevated heating temperature in a sealed tank are considered as big drawbacks of the solvothermal synthesis techniques

### 3.2. Ionothermal Process

In the ionothermal process, ionic liquid/molten salt, i.e., ZnCl_2_, have been explored for COFs synthesis, for example, CTF-1, CTF-2. A thick-walled tube, such as a Pyrex tube, as well as elevated heat (400 °C) and pressure, are the ionothermal conditions necessary for COFs synthesis. In this type of synthesis process, they served as both a solvent and an accelerator. In the last step, ZnCl_2_ is removed from the resultant CTF mixture and is then cooled to room temperature, crushed, and completely rinsed with water [22]. The powder is then agitated for 15 h in diluted HCl to eliminate ZnCl_2_, then filtered, washed with water and THF, and finally dried under vacuum. Likewise, ionic liquid as a solvent has provided a convenient, moderate, and green chemistry method for 3D COF synthesis. For example, 3D-IL-COF-1 may be achieved in three minutes, indicating a fast reaction process using ionic liquid. Through this approach, a series of 3D ionic-liquid-comprising COFs has been successfully synthesized. In addition, the ionic liquid was discovered to be reusable, with acceptable activity loss of crystalline materials. Such type of research not only introduces a novel synthesis method for COFs, but also paves the way for green large-scale COF production in industry [24].

### 3.3. Microwave Assisted Synthesis

To counter the limitation of solvothermal synthesis techniques, the microwave assisted synthesis approach has been investigated and reported for the fast production of crystalline porous COFs. Many COFs, i.e., boronate-ester-linked COF-5, 2D COF-5, COF-102, and 3D COF-102, have been reported using microwave-assisted synthesis. Usually, twenty minutes was needed for COF synthesis using microwave irradiation, which is significantly quicker than the 72-h prolonged reaction time necessary under solvothermal synthesis conditions. Furthermore, microwave heating has been shown to improve the properties of COFs, such as the surface area. For example, a 2019 m^2^ g^−1^ BET surface area was found in the case of microwave-assisted synthesis techniques, which is somewhat greater than that for the solvothermal synthesized method. Apart from this, microwave solvent extraction methods have many advantages, such as removing copolymers from COFs more efficiently, producing COFs with high permeability, etc. [24].

### 3.4. Mechanochemical Synthesis

Since both microwave and solvothermal processes are performed under difficult circumstances (e.g., reaction in a sealed Pyrex tube, inert environment, appropriate solutions, pressure and temperatures for crystallization, etc.), a straightforward synthesis technique is needed for COF synthesis. Mechanochemical synthesis is a synthesis technique which fulfil these needs. Essentially, in mechanochemical synthesis, the monomers are placed into a mortar and crushed with a pestle at ambient temperature to produce COFs. A small quantity of catalyst solution is transferred to the mortar during crushing the subunits, which improves the reagent’s effectiveness. Furthermore, ketoenamine, imine, and hydrazine COFs were synthesized using mechanochemical synthesis [22,23]. It has been reported that ketoenamine COFs were produced in excellent quality with a high specific surface area by crushing the diamine molecules with p-toluenesulfonic acid with a small amount of water, then adding TPG under 170 °C heating for one minute. Despite the advantages of being quick and simple, mechanical grinding of COF precursor reactants typically results in either amorphous or weakly crystalline structures [23]. Straightforward, cost-effective, and environmentally friendly approaches are many remarkable advantages of the mechanochemical COF synthesis.

### 3.5. Sonochemical Method

The sonochemical method is another approach to handle the limitations of solvothermal synthesis. Clouds are produced in the solution using ultrasound, which develop and burst in a process known as cavitation, resulting in an extremely high temperature and pressure in the solution, thereby speeding up the rate of the chemical process [22,24]. For instance, COF-1 and COF-5 reactions may be increased up to a 0.5 L batch size after a quicker reaction time of just 0.5–2 h, resulting in a BET surface area of up to 2122 m^2^ g^−1^ [9,21,22]. However, the sonochemical synthesis method has been reported only for a limited number of COF syntheses.

### 3.6. Vapour-Assisted Method

More recently, thin COF films have been reported based on the vapor-assisted COF synthesis method. For example, COF precursors were mixed in appropriate solvent mixture, such as acetone and ethanol, and then drop-casted on a substrate, which was subsequently placed in a desiccator with a reservoir mixture containing a 1:1 ratio of mesitylene and dioxane. A smooth, homogenous COF film was prepared after 72 h at ambient temperature. As a result, the crystallinity of the products was found under strongly influenced solvent reservoirs. The thin film growth was proved on a variety of surfaces, with a variety of COFs, and with the ability to adjust the depth of the layer by altering the quantity of water [23,24].

### 3.7. Light Promoted Synthesis

Utilizing the Sun’s light/energy has been reported as the most recent synthetic method for COF synthesis. It was observed within three hours of reaction time, light promoted extremely covalently linked and crystalline materials of COFs. For example, COF (hcc-COF) was synthesized using a mixture of hexaketocyclohexane octahydrate (HCH) and 1,2,4,5-benzenetetramine tetrahydrochloride (BTA) (in different solvents) in a quartz tube in an atmosphere of air under simulated visible light of 200–2500 nm wavelength. To ensure effective imine condensation, a small quantity of water and acetic acid were mixed as cocatalysts. In luminous circumstances, it was discovered that COF existed. The synthesized hcc-COF has the greatest electrical properties of 2.22 × 10^−3^ Sm^−1^, owing to its extended conjugated framework, which aids electron transport [10,24]. Table 2 describes different COFs, its synthesis methods and suitable conditions for its synthesis.

**Table 2:** COF type, synthesis method and favorable conditions for its synthesis.

S. No.	COF	Synthesis Method	Monomers	Reaction Conditions	References
1.	COF-5	Microwave assisted	BDBA + HHTP	1,4-Dioxane/mesitylene, 100 °C, 20 min	[24]
2.	COF-102	Microwave assisted	TBPM	1,4-Dioxane/mesitylene, 200 W, 100 °C, 20 min	[24]
3.	TpPa-COF	Microwave assisted	Pa + Tp	1,4-Dioxane/mesitylene, acetic acid, 100 °C, 60 min	[25]
4.	TfpTP-H	Microwave assisted	TP-H + Tp	DCE/1,4-dioxane, acetic acid, 250 W, 175 °C, 10 min	[26]
5.	TfpTP-OEt	Microwave assisted	TP-OEt + Tp	DCE/mesitylene, acetic acid, 250 W, 175 °C,15 min	[26]
6.	TfpTP-OMEG	Microwave assisted	TP-OMEG + Tp	DCB/prop, acetic acid, 250 W, 175 °C, 20 min	[26]
7.	TfpTP-ODEG	Microwave assisted	TP-ODEG + Tp	DCB/prop, acetic acid, 250 W, 175 °C, 20 min	[26]
8.	CTF-1	Ionothermal	DCB	ZnCl2, DCB, 120–210 W, 400 °C, 0.5–2 h	[24]
9.	TpBD	Room temperature synthesis	Tp + DABP	EtOH, RT for 30 min	[27]
10.	COF-LZU-1	Room temperature synthesis	BTCA + Pa	1,4-dioxane, acetic acid, RT, 72 h	[28]
11.	TpPa-1	Room temperature synthesis	Tp + Pa	1,4-dioxane, acetic acid, RT, 72 h	[28]
12.	N_3_–COF	Room temperature synthesis	HZ + TFPT	DCE/EtOH, acetic acid, RT, 72 h	[28]
13.	TFPB-HZ	Room temperature synthesis	HZ + TPB	EtOH/acetic acid, RT, 72 h	[28]
14.	CAF-1	Aqueous phase-mediated synthesis	TMA + CHDA	DMF/H_2_O, 250 °C, 72 h	[29]
15.	CAF-2	Aqueous phase-mediated synthesis	MTAB + CHDA	DMF/H_2_O, 250 °C, 72 h	[29]
16.	HCOF-2	Aqueous phase-mediated synthesis	B1 + HZ	H_2_O, 120 °C, 12 h	[30]
17.	TpPa-1 (MC)	Mechanochemical synthesis	Tp + Pa	1,4-Dioxane/mesitylene, 45 min	[31]
18.	TpPa-2 (MC)	Mechanochemical synthesis	Tp + MPA	1,4-Dioxane/mesitylene, 45 min	[31]
19.	TpBD (MC)	Mechanochemical synthesis	Tp + DABP	1,4-Dioxane/mesitylene, 45 min	[31]
20.	TpBD-Me_2_	Mechanochemical synthesis	Tp + DABP-Me_2_	PTSA-H_2_O, 170 °C, 60 s	[32]
21.	TpAzo	Mechanochemical synthesis	Tp + Azo	PTSA-H_2_O, 170 °C, 60 s	[32]

## 4. Biological Applications of COFs

We have found that COFs are promising materials for catalysis, sensing, optoelectronics, small molecules adsorption, gas storage and separation, and drug delivery. This is due to the favorable characteristics of COFs, i.e., an inherent permeability, excellent pore aperture, large surface area, ordered channel structure, low density, high dimensionality, large size, and tunable pore compatibility. In this section, we critically discuss COFs for biological applications using recently reported research.

### 4.1. Drug Delivery

**Figure 9:**
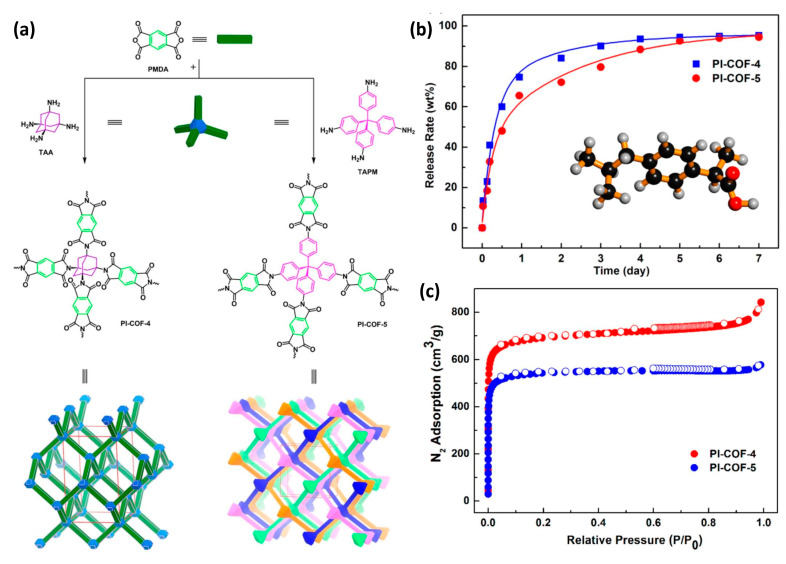
(**a**) Synthesis and structural representations, (**b**) BET surface area, and (**c**) drug release profile of PI-COF-4 and PI-COF-5 with ibuprofen (IBU) as a model drug release in a simulated body [33].

When compared to MOFs, COFs are still in early developmental stages for drug delivery applications; however, it is obvious that the use of COFs for therapeutics is gaining attraction, and significant attempts have been undertaken to develop COF-based therapeutics [10]. The very first attempts to employ COFs for pharmaceutical applications were carried out using 3D polyimide COFs, i.e., PI-COF-4 and PI-COF-5 with excellent thermal properties and surface territory [33]. The imidization process was used to combine tetrahedral and linear key components to create PI-COFs. For drug delivery applications, both COFs had high drug loading and excellent drug release profiles. When the tetrahedral key components, i.e., tetra(4-aminophenyl) methane (TAPM) and 1,3,5,7-tetraaminoadamantane (TAA) were combined with the linear connecting unit, pyromellitic dianhydride (PMDA), the extended 3D framework configurations PI-COF-4 and PI-COF-5 were formed (Figure 9) [33]. These COFs were filled with an anti-inflammatory chemical, such as IBU, which had a drug loading capacity, and a 6-day release of 24% and 20%, respectively.

Furthermore, as compared to PI-COF with a reduced pore diameter, the release rate was lower (60% for PI-COF-4 vs. 49% for PI-COF-5 after 12 h), indicating that pore size and geometry are directly connected to drug delivery in COFs [33]. Additionally, adding functionality to COFs resulted in improved drug delivery. This was observed in 8-hydroxyquinoline functionalized COF (COF-HQ). The COF-HQ can be utilized as a carrier to enhance drug loading by adding the quinoline group, since it is simple to protonate the nitrogen element on the conjugated heterocycles (quinoline) because it possesses a lone pair of electrons with high activity [34]. The list of different COFs has been listed in Table 3 given below.

### 4.2. Photodynamic (PDT) and Photothermal Therapy (PTT)

Traditional chemotherapy methods suffer from poor efficacy, a high cost, many adverse effects, etc. To overcome all these limitations, phototherapy is a potential treatment method when compared to other cancer therapeutic techniques. It has garnered a lot of interest due to its various advantages, including a high efficacy, low cost, little invasiveness, minimal adverse effects, etc. The use of COF photodynamic and photothermal therapy adds many more advantages, due to their structural and physiochemical features. They result in regular pores with large diameters, allowing large-sized molecules, such as porphyrins and phthalonitriles, to be encapsulated more easily. Moreover, COFs are metal-free analogues of porous materials and should be extremely biocompatible; they might also avoid the possible toxicity of metal containing nanomaterials [11].

**Figure 10:**
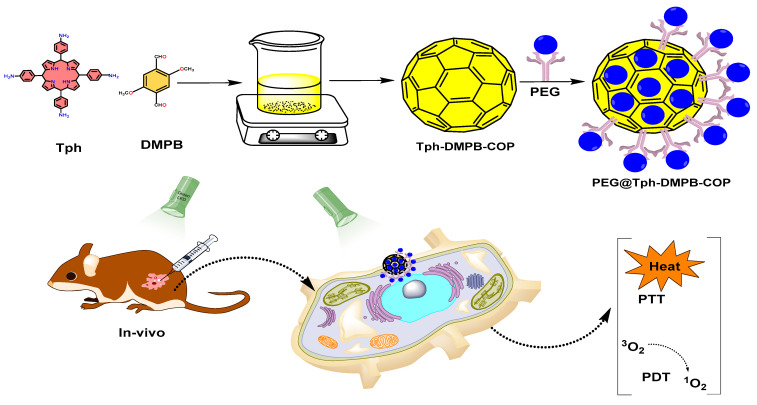
Schematic representation of a PEG-protected COF synthesized from Tph and DMTP, and its PDT and PTT applications [48].

In recently reported work, nanoscale COFs (NCOF) can be employed as a dual modal (PDT/PTT treatment) platform when synthesized using a simple synthetic method in ambient circumstances. For example, stepwise BDF and guest encapsulation techniques were employed for the successful synthesis of VONc@COF-Por. In this report, PDT and PTT effects were seen separately in covalently grafted porphyrinic PS (Por) and noncovalently loaded naphthalocyanine PTA (VONc). It showed strong ^1^O_2_ production and a photothermal conversion capacity of 55.9% when exposed to visible light [10]. Furthermore, when imine-linked COFs were placed on the surface of Fe_3_O_4_ nanostructures for PTT, the findings indicated that Fe_3_O_4_@COF had a 21.5% photothermal conversion efficiency, which was similar to that of other well-studied photosensitizers, such as Au nanostructures [47]. Additionally, Tph–DMTP–COP nanoparticles were also produced at ambient temperature via a Schiff base condensation process. Under 650 nm laser irradiation, porphyrin functionalized COF produced an extended conjugated arrangement with strong π–π stacking interactions, leading to an outstanding photothermal effect with a 34.88% photothermal conversion efficiency. The synergistic effect of PDT and PTT both in vitro and in vivo resulted in an increased anticancer effectiveness. Because of its low toxicity, strong biocompatibility, great photodynamic effect as well as photothermal effect, and enhanced tumor suppression effectiveness, this porphyrin-based COP might be employed as both a photothermal agent and a photosensitizer in cancer phototherapy [48]. The complete schematic representation, from the synthesis to PDT and PTT applications, is given in Figure 10.

Another porphyrin-containing covalent organic polymer (PCOP) synthesized through a room temperature solution-based ageing technique showed enhanced anticancer effects. The resultant nanoparticles had a high photothermal conversion efficiency (21.7%), as well as a good photodynamic effect. These results indicate that COFs are very promising emerging materials in biological applications. In the future, it is hoped that more functioning COPs will be developed and employed in a variety of biological applications [49].

### 4.3. Biosensing and Bioimaging

**Figure 11:**
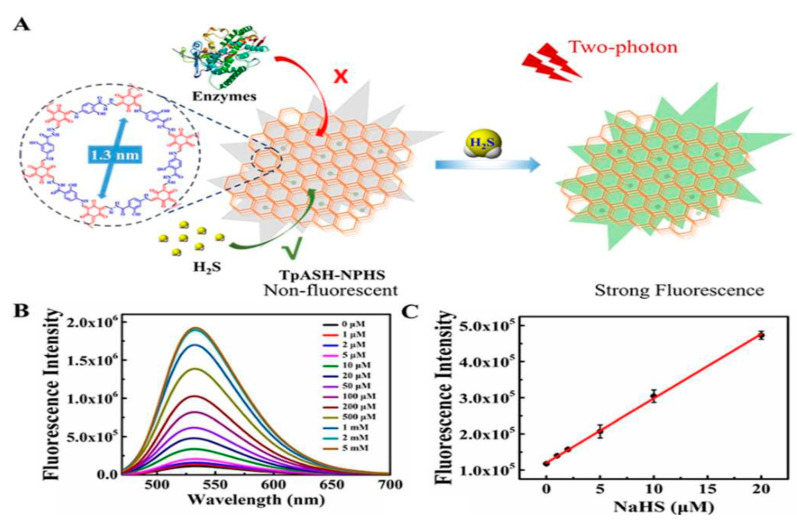
(**A**) H_2_S action on TpASH-NPHS; (**B**,**C**) fluorescence intensity at different concentrations and the correlation curve [50].

The COFs showed unique properties such as good porosity, large diameter, increased BET surface area, π-π stacking, high hydrogen bond interaction with biomolecules and excellent host–guest interactions and, hence, they are explored for biosensing and bioimaging applications. Hopefully these features will have real-world utility in the near future for diagnosis and treatment. For instance, TpASH-NPHS, a two-photon fluorescent COF nanostructure, exhibits excellent chemical and photostability. It was reported that TpASH-NPHS has a highly selective and sensitive fluorescence sensor for gas transmitter hydrogen sulphide (H_2_S) detection (Figure 11) [50]. Importantly, COFs exhibit efficient detection and imagining of H_2_S in live cells and deep tissues, decreasing the cellular fluorescence intensity, minimizing tissue damage, and improving the tissue absorption rate under UV stimulation [50].

Furthermore, a graphene-based two-dimensional single-layered nanomaterial has also been reported with good stability, more functionality, and an appropriate band gap for excellent bioselectivity and bioimaging properties. For instance, g-C_3_N_4_ with a high condensation of carbon nitride 2D COF of tri-s-triazine or s-triazine linked via tertiary amines results in a metal-free graphite-like layered structure. It exhibits an appropriate band gap of approximately 2.7 eV and a layer spacing of around 0.33 nm [51].

In 2014, researchers used an imine-based COF thin film with a silicon substrate functionalized with an amine for BSA protein and DNA electrochemical detection in aqueous solution [52]. BSA protein was absorbed on the imine-functionalized 2D COF thin surface through electrolytic contact after electrolysis. Unlike typical surface processes, this absorption increases the electrochemical performance of the BSA tagged COF thin films composite, resulting in a decrease in the simulated charge-transfer resistance (Rct) value evaluated by electrochemical impedance spectroscopy (EIS). Thus, by utilizing the BSA tagged 2D COFs and EIS-based calculation of the decrease in Rct value, BSA protein or DNA may be detected in aqueous solution [52].

## 5. Miscellaneous Applications

### 5.1. 3D Printing Technology Applications

**Figure 12:**
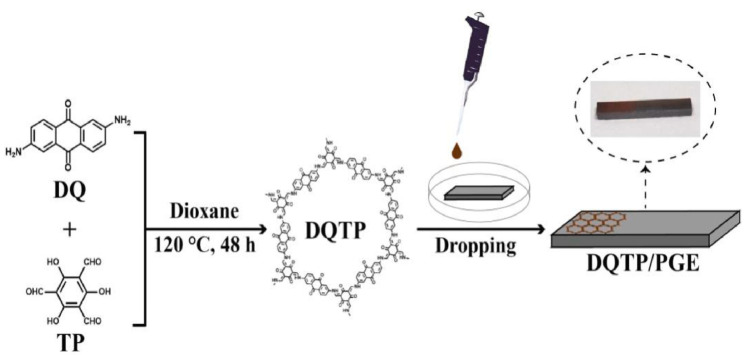
Graphical illustration for a 3D-printed electrochemical electrode clamp based DQTP/PGE for the simultaneous determination of bisphenol A and bisphenol S [54].

**Figure 13:**
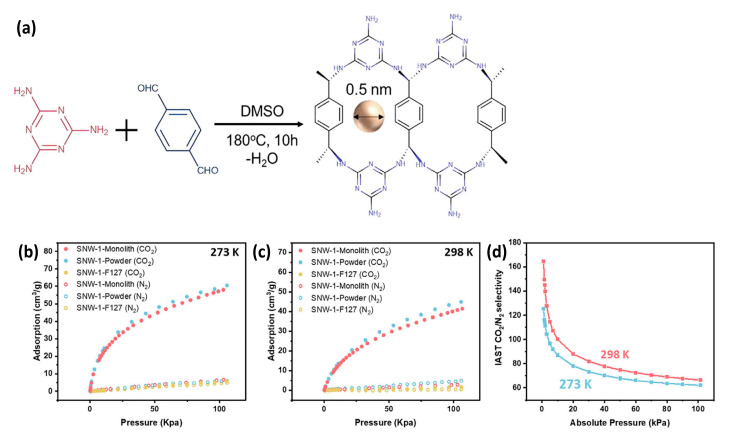
(**a**) SNW-1 COF monolith synthesis procedure using melamine and terephthalaldehyde; (**b**) CO_2_ and N_2_ adsorption curve of SNW-1 monolith, SNW-1 powder, and SNW-1/F127 monolith at (**b**) 273 K and (**c**) 298 K; (**d**) sorption selectivity of CO_2_ over N_2_ SNW-1 monolith by IAST calculation [55].

In 2021, Mallakpour et al. reported a literature study on the applications of 3D printing technology in water treatment, biomedicine, the electronic industry, and gas removal using metal organic framework (MOF)/covalent organic framework (COF) based materials [53]. Afterward, many researchers reported some initial research reports on 3D printing technology applications using these materials. For example, Huang et al. investigated a portable 3D-printed electrochemical electrode clamp based on a 2,6-diaminoanthraquinone (DQ) and 1,3,5-triformylphloroglucinol (TP) modified pencil graphite electrode (DQTP/PGE), Figure 12 [54]. The β-Ketoenamine-linked COF DQTP was prepared through a solvothermal method to achieve high porous crystallinity, with excellent conductivity for bisphenol A and bisphenol S determination [54]. A 3D-printed electrochemical electrode clamp-based sensor exhibited good linearity (0.5–30 μM range) for two bisphenols with a sensing limit of 0.15 μM (S/N = 3), Figure 13 [54].

A DQTP/PGE-based sensor showed a stable response and good reproducibility with real food packaging samples over a specific time period. In another direction, Liu et al. investigated a binder-free 3D-printed Schiff-base network-1 (SNW-1) monolith for CO_2_ adsorption (Figure 13a), exhibiting undiminished BET surface area, porosity, and free surface functionalities when compared to its powder-form analogue, which provide its enhanced CO_2_ adsorption and CO_2_/N_2_ sorption selectivity (Figure 13b). Importantly, CO_2_ uptake of the SNW-1 monolith at 1 bar were found to be 58.42 cm^3^ g^−1^ and 41.41 cm^3^ g^−1^ at 273 K and 298 K, respectively. Overall, this research report is a first key step in binder-free 3D printable formulation using COF monoliths for high CO_2_ adsorption [55].

More recently, Bulit et al. reported a new prototype for the synthesis and processing of COFs. This research group convey the combination of microfluidic technologies with 3D printing technology. Therefore, direct-ink writing (DIW) and 3D printing are two other methods that could help COFs handle more information and exhibit more advantages [56]. We hope that such a type of process adaptation will be well-matched with microfluidic conditions and able to achieve functional 3D COF materials for diverse applications.

### 5.2. Gas Storage and Transport

In the present literature, high porosity and crystallinity of COFs have attracted great attention as promising materials for gas storage and separation of essential gases, such as hydrogen, CO_2_, and methane. For example, 3D-COF, i.e., COF-102, has been reported with a high BET surface area of 4650 m^2^ g^−1^ and demonstrated a greater CH_4_ storage capacity of 25 wt% (203 cm^3^/cm) compared to the 15 wt% capacity of COF-320 at 298 K and 80 bar [57]. Likewise, doped 2D-COF-1 with pyridine atoms between the level layers exhibited a higher hydrogen absorption limit [58]. Further, in another report, ACOF-1, a novel azine-linked COF, was synthesized via a solvent evaporation reaction of hydrazine hydrate with 1,3,5-triformylbenzene at a temperature of 273 K and a pressure of 1 bar. ACOF-1 has a greater surface area and a tiny void distribution. Therefore, it absorbs up to 9.9 mg g^−1^ H_2_, 177 mg g^−1^ CO_2_, and 11.5 mg g^−1^ CH4, with stronger selectivity towards carbon dioxide than nitrogen and methane [59]. COF-TpAzo, another COF with high gas storage capability, displays strong selectivity in CO_2_/N_2_ due to the COF’s N_2_-phobic and CO_2_-philic properties at 1 bar. Finally, using COF-TpAzo, the storage capacities of 10.6 mg g^−1^ for H_2_ and 11.2 mg g^−1^ for CH_4_ were also reported [60].

### 5.3. Solar Cells

The massive consumption of non-renewable fossil fuels results in serious environmental problems and energy crises. Hence, for promoting a sustainable environment, clean and environmentally friendly energy storage technologies and energy conversion systems are urgently needed to address the global energy demand and environment issues [61,62,63,64]. Solar energy is an inexhaustible and clean energy source which can be effectively trapped by a popular tool called a solar cell. A solar cell is a device that utilizes the Sun’s energy and converts it into electrical energy [61,62,63,64]. For attaining higher energy conversion efficiency and energy densities, novel and versatile materials possessing a high performance are required.

COFs possess a high crystalline and conjugated properties with a high dimensional geometry. They have been extensively explored in photovoltaic device applications, including dye-sensitized solar cells (DSSCs) and perovskite solar cells (PVSCs). Both 2D and 3D COFs can be used in photovoltaic (PV) applications; however, 3D COFs are less reported in contrast to 2D COFs. Jiang and co-workers reported a chemically stable and photoconductive fullerene-loaded CS-COF, synthesized by the co-condensation of tert-butylpyrene tetraone (PT) and triphenylene hexamine (TPHA), possessing a 0.9% power conversion efficiency with an open circuit voltage of 0.98 V [61]. Later, Bein and co-workers implemented a thiophene-based COF by co-condensing hexa-hydroxytriphenylene and thieno-[3,2-b]-thiophene-2,5-diyldiboronic acid, showing a PCE of up to 0.05% with an open circuit voltage of 0.62 V in DSSCs [62]. Recently, a highly conjugated 3D COF based on spirobifluorene as a building unit with amine bonds has been implemented in PSCs. Simple doping of the prepared COF as an additive in PV devices can be achieved, in which the PCE was improved by 15.9% and 18.0% for SP-3D COF-1 and SP-3D COF-2, respectively, in comparison to the reference undoped solar cell sample [63]. A 2D COF with a core building unit of tetraphenyl ethylene and linkages of carbazole and pyrene have shown remarkable a increase in PCE. Additionally, when pyrene and carbazole are attached to 4,4′,4″.4‴-(ethane-1,1,2,2-tetrayl) tetranilino (ETTA) obtained by a solvothermal process, this results in TFPPy-ETTA-COF and Car-ETTA-COF. Both COFs showed a high crystallinity, thermal stability, and well conjugated properties. It has been reported that the PCE for Car-ETTA-COF was 19.79%, while in the case of TFPPy-ETTA-COF, it was 19.72% [64].

In another study, polyimide COFs (PI-COFs) were doped in TiO_2_ as a photoelectrode in a dye-sensitized solar cell (DSSC), in order to operate with N719 dye. Under conditions that simulate one sun illumination, an unadulterated DSSC that has not been doped with PI-COFs, has an efficiency of 9.05% in the conversion of electrical power. The short-circuit current density (JSC) is increased by 0.04 weight percent, from 17.43 to 19.03 mA/cm^2^, and the cell efficiency is increased (9.93%). The increase in JSC is due to the PI-COF’s bifunctionality, which improves charge transfer/injection and inhibits charge recombination through the interaction between PI-COF and N719 dye. PI-COFs also serve as a co-sensitizer and supply a modest amount of photoinduced electrons when exposed to sunshine. The hydrophilicity of PI-COF particles is increased, and the heterogeneous coupling between PI-COF and TiO_2_ nanoparticles is strengthened, leading to a more effective charge injection when the oxygen plasma’s surface is modified. As a result, an improved JSC of 19.43 mA/cm^2^ and a power conversion efficiency of 10.46% were observed. This approach to improving the solar efficiency involves doping TiO_2_ nanoparticles with PI-COFs to modify the photoelectrode, which has the potential to further the development of DSSCs [65].

In this study, two fluorinated side chain conjugated polymers known as P1 (2 F) and P2 (4 F) in tandem polymer solar cells (TPSCs) were investigated. The enhanced absorption coefficients and deep highest occupied molecular orbitals of these conjugated polymers are what cause their high open-circuit voltages (Voc), which are 0.91 and 1.00 V, respectively. P1 or P2 front cells and PTB7-Th rear cells have been effectively combined to create TPSCs using these conjugated polymers. With impressive power conversion efficiencies of 11.42 and 10.05% and high Vocs of 1.64 and 1.72 V, respectively, the improved TPSCs provide excellent power output. By balancing charge mobilities to assess these findings and combining optical and electrical modelling, it is possible to show that all the photovoltaic characteristics in TPSCs have improved simultaneously [66].

### 5.4. Super-Capacitors

Among all energy storage technologies, super-capacitors have drawn attention because of their typical long cyclic stability and high energy density. According to their charge storage mechanism, super-capacitors can be classified as pseudo-capacitors or electrochemical double-layer capacitors. EDLCs can store charge without electron transfer between the electrode and electrolyte interfaces, while in the case of pseudo-capacitors, energy is stored by a reversible, instant intercalation course and a fast-redox reaction with electron transfer between the electrode and electrolyte.

In super-capacitors, charge storage occurs when fast absorption/desorption takes place. The application of COFs in super-capacitors has shown advantageous features, such as (A) a controllable pore structure and high surface area, which can provide room for an electrolyte (in EDLCs); and (B) redox-active functionalities are appropriate for pseudo-capacitive energy storage. Dichter and co-workers were the first who introduced two 2D COFs to super-capacitor applications [67]. Another two chemically sTable 2D COFs with ketoenamine linkages are DAAQ-TFP COF and DAB-TFP COF. It has been reported that the efficiency of super-capacitors can be increased by doping nitrogen and carbon materials, as it offers a pseudo-capacitance effect [68]. Gu et al. obtained a highly conductive 2D COF named Ni-COF with a high capacitance of 1257 Fg^−1^ [69]. In another report, aza-π conjugated COFs, a particular class of permeable organic solids, show great conductivity because of broadened 2D aromatized π-conjugated linkages with π-π overlapping, thereby contributing a consistent path for the electrons to bring about the leading material [70]. A study on 2D COF synthesized by a solvothermal method showed a capacitance of 163 Fg^−1^ at 0.5 Ag^−1^ and at a voltage of 0–2.5 V [71,72]. In another report a PDC-MA-COF super-capacitor produced by an aldehyde amine condensation reaction displayed an enhanced energy density of 29.2 W hkg^−1^ and a force thickness of 750 Wkg^−1^. Additionally, it showed brilliant cyclic stability and could hold 88% capacitance after 20,000 charge–discharge cycles.

### 5.5. Photocatalysts

The construction and adjustment of photocatalysts exhibiting excellent efficiency, depending on the usual photocatalytic activity, would include factors such as increased visible-light absorption capacity, easier electron–hole splitting, and reduced photo-corrosion over longer periods of time. COFs as a versatile substrate are featured prominently in photocatalysis, and many techniques for increasing their activity based on modified COFs have been devised. The following steps can be taken to improve visible-light absorbance and minimize electrons and hole recombination. The most direct path is to incorporate a relevant functionality to COF. The structural elements from which COF is constructed affect its physiochemical properties, allowing structural alteration of their energy band arrangement. Furthermore, elemental doping can serve as another simple and effective approach to adjust the chemical and physical properties of COFs at the nanoscale. An alternative strategy for improving COFs’ photocatalytic activity is to add either an appropriate chromosphere or a suitable sensitizer. All of the aforementioned methods make use of solar energy throughout a broad spectrum, which is vital for photocatalytic reactions [73].

Fuel scarcity is currently one of the hardest challenges to address, especially in a safe and renewable manner. Hydrogen evolution by UV illumination is the foremost important renewable energy source. In 2014, the very first application of a COF for photocatalytic hydrogen generation was discovered. Due to the high electrical conductivity and electron-withdrawing properties, the triazine-based structure showed high performance for hydrogen evolved reactions. For instance, coupling 2,5-diethoxy terephthalohydrazide (DETH) with 1,3,5-tris-(4-formyl-phenyl) triazine (TFPT) gives crystalline hydrazone-linked COF (TFPT-COF). The pore size of TFPT-COF was found to be 3.8 nm, which the largest among all the hydrazone linked COFs. Furthermore, the H_2_ evolution property can be enhanced by substituting ascorbic acid with triethanolamine (TEOA) as an electron donor, though with the compromise of a faster deactivation of the COF. With the addition of 10 vol% TEOA, the H_2_ evolution rate was found to be 1970 μmol h^−1^ g^−1^ corresponding to a quantum efficiency of 2.2%. This rate was found to be enhanced approximately three times when compared with standard photocatalytic systems, such as Pt-modified amorphous melon, crystalline poly (triazine imide), and other carbon nitrides, [73,74,75]. In another report, the solid, planar dibenzo thiophene sulfone (DBTS) subunit was found to be favorable to light-induced photocatalytic evolution. To explore the catalytic performance, the DBTS unit was integrated into the designed COFs. The hydrogen evaluation rate of the as-prepared FS-COF was up to 16,300 mmol h^−1^ g^−1^, which is about 10% larger than that of the N_3_-COF. COF-based photocatalysts for hydrogen production have also been produced by modifying the fundamental components and connections. For example, by expanding alkynes with the alteration of peripheral heteroaromatic building blocks, a series of planar pyrene-based COF (A-TEXPY-COFs) was synthesized. In this system, the most advanced donor has the highest H_2_ evolution rate of 98 mmol h^−1^ g^−1^ [76].

Photocatalytic water oxidation research with COF photocatalysts is considerably less developed than photocatalytic H_2_ production research, as the breakage of the O–H bond, the creation of the O–O bond, and the high overpotential with slow O–O formation kinetics are all involved in water oxidation in the case of oxygen evolution, which is more complex, with the rate-determining step (four-electron redox process) in total water splitting. Recently, photocatalytic water separation has been discovered using covalent triazine-based frameworks (CTFs), a class of semi-conductive materials. Their tunable band gap and easy fabrication provide them with many possibilities for research and development. CTF-1, for example, was produced via microwave-assisted condensation at various powers and was subsequently used in water splitting. Under similar photocatalytic circumstances, the material (CTF-0-M2), produced using the microwave technique, has an excellent photocatalytic activity for H_2_ production (up to 7010 mol h^−1^ g^−1^). This is about seven times consistently higher than the other framework (CTF-0-I) created using traditional ionothermal trimerization. After seven cycles in visible light, this results in a higher turnover number (TON) of 726 for the Pt cocatalyst. The material produced via the ionothermal technique, on the other hand, is the most efficient in releasing oxygen. Under similar experimental circumstances, CTF-0-I initially generates approximately six times more oxygen gas than CTF-0-M2. The quantum efficiency of CTF-0-I is evident. CTF-0-I produces oxygen with an obvious quantum efficiency (AQY) of 5.2% at 420 nm in the absence of a cocatalyst [77].

With the world’s population and economy expanding at a rapid pace, increased coal and oil use has resulted in increased carbon dioxide (CO_2_) emissions, causing significant environmental issues such as the greenhouse effect, temperature increase, climate change etc. Photocatalytic CO_2_ reduction to clean renewable resources has raised attention in recent years as a promising approach for simultaneously tackling environmental and energy concerns. COFs, the potential photocatalytic candidates, are acknowledged as a spectacular platform for photocatalytic CO_2_ reduction due to their strong CO_2_ absorption capacity and specificity.

Azine-based COFs containing p-stacking aromatic units are considered as the most promising photocatalytic possibilities. The separation and transmission of photo-induced electrons/holes might be aided by a large, conjugated structure. Two azine-linked crystalline COFs, such as ACOF-1 (hydrazine, TFB) and N_3_-COF, were recently used as photocatalysts for UV-induced CO_2_ reduction using H_2_O as a hole scavenger. The large surface area of ACOF-1 (1053 m^2^ g^−1^) and N_3_COF (1412 m^2^ g^−1^) in this work, along with numerous accessible nitrogen sites, resulted in strong CO_2_ absorption, facilitating the photocatalytic reduction of carbon dioxide to methanol. The total quantity of CH_3_OH produced over N_3_-COF after 24 h of visible light irradiation was 13.7 mmol g^−1^, which was more than ACOF-1 (8.6 mmol g^−1^). In comparison to ACOF-1, N_3_-COF, with electron-poor triazine groups, can maintain the negative charge produced on the COF. This is critical for improved photocatalytic activity [73,78,79].

## 6. Conclusions

This review has discussed the significant developments in biomedical applications using COFs, due to their numerous advantages, such as a high surface area, a porous structure, exceptional modification ability, etc. However, despite such desirable attributes, COFs still face many issues in being applied for successful practical biomedical applications, i.e., hydrolytic stability, targeted delivery, biocompatibility, challenges in synthesis techniques, and so on. In a real sense, they tend to decline the success rate for obtaining fruitful results. As a result, it becomes significantly important to address the issues via extensive research to overcome the limitations of COFs as far as biomedical purposes are concerned. Furthermore, the integration of functional groups to the synthesized COFs enhances their biological applications. However, tagging functional groups on the surface of COFs is still challenging due to poor stability and other unfavorable conditions.

Secondly, there is an urgent need for novel synthesis strategies to produce large numbers and quantities of COFs for COF technology in the near future. Because most COF synthesis processes involve condensation reactions between the same or different organic ligands in a suitable solvent ratio, this hinders large-scale COF manufacture. To overcome this limitation, room-temperature and solvent-free synthesis processes must be developed for the large-scale manufacturing of COFs. Moreover, understanding the relevance of reversible bond formation has been vital in the development of novel COFs and enhancing their crystallinity, although determining their structure remains a challenge. The structure and many other key characteristics of COFs, such as bond connectivity, the nature of bonds, the position of atoms, and the location of guest molecules remains inaccessible. Recently, several researchers succeeded in synthesizing large crystals of COFs by a slow crystallization process. This enables us to understand the COFs’ structure in a more detailed manner via single-crystal X-ray diffraction analysis.

COFs exhibit unique properties that allow them to be used in a variety of fields, such as catalysis, energy storage, gas storage and separation, optoelectronics, and so on. Their advancements offer more opportunities for researchers in the near future. When compared to other porous materials, COFs offer several unique applications. Post-synthetic modifications further improve the conductivity and electrocatalytic activity of COFs. Importantly, COFs can also be used as a precursor in the development of carbon nanomaterials containing a single metal or metal clusters, which can offer excellent electrocatalytic performance. The cost issues and synthetic challenges, such as morphology and size control, discourage COFs materials from being used as precursors in making carbon materials; however, using COFs as precursors to prepare single-atom catalysts (SACs) could be an interesting path to pursue. When considering industrial applications, the cost is a crucial concern; therefore, by making COF materials scalable and by achieving functional criteria, such as long span stability, reversibility, processability, and high performance.

Moreover, most of the research on COF involves imine, boroxine, hydrazine, and cyanide linkages. Very few COF synthesis reactions contains peptide linkages (condensation between acid and imine bonds). Efforts must be made by researchers to synthesize COFs based on acid–base reactions. Hopefully, COFs will gain a foothold in the industrial field soon.

## Figures and Tables

**Table 1:** Topology design diagram, condensation type, topology type and monomer symmetry of recently reported COFs.

S. No	Topology Design Diagram	Condensation	Monomer Symmetry	Topology Type	Reference
1.	[C_3_ + C_2_] design	Poly-condensation	C_3_ and C_2_ symmetric monomers	Hexagonal	[16]
2.	[C_4_ + C_2_] design	Poly-condensation	C_4_ and C_2_ symmetric monomers	Tetragonal	[16]
3.	[C_2_ +C_2_] design	Self-condensation	C_2_ symmetric monomers	Rhombic	[16]
4.	[C_6_ + C_2_] design	Poly-condensation	C_6_ and C_2_ symmetric monomers	Triangular (microporous)	[16]
5.	[C_3_ + C_3_] design	Self-condensation	C_3_ symmetric monomers	Hexagonal (microporous)	[17]
6.	[C_2_ + C_2_] design	Self-condensation	C_2_ symmetric monomers	Hexagonal (microporous)	[17]
7.	[C_4_ + C_4_] design	Self-condensation	C_4_ symmetric monomers	Tetragonal (microporous)	[17]
8.	[C_3_ + C_1_] design	Poly-condensation	C_3_ and C_1_ symmetric monomers	Hexagonal	[18]
9.	[T_d_ +T_d_] design	Self-condensation	T_d_ monomers	Dia	[18]
10.	[T_d_ + C_3_] design	Poly-condensation	T_d_ and C_3_ symmetric monomer	bor, ctn, srs	[18]
11.	[T_d_ + C_2_] design	Poly-condensation	T_d_ and C_2_ symmetric monomer	Dia	[19]
12.	[T_d_ + C_4_] design	Poly-condensation	T_d_ and C_4_ symmetric monomer	Pts	[19]

**Table 3:** List of newly reported COFs for biological applications.

S. No.	Covalent Organic Framework	Organic Linkers	Therapeutics	Drug Loading	Drug Release	Reference
1.	Polyimide-covalent organic framework-4 PI-COF-4	Pyromellitic dianhydride (PMDA) + Tetrahedral 1,3,5,7-tetraaminoadamantane (TAA)	IBU (ibuprofen)	24 wt%	60% after 12 h	[33]
2.	Polyimide-covalent organic framework-5 PI-COF-5	Pyromellitic dianhydride (PMDA) + Tetra (4-aminophenyl) methane (TAPM)	IBU (ibuprofen)	20 wt%	49% after 12 h	[33]
3.	t PEG-CCM@APTES-COF-1	Polyethylene-glycol-modified monofunctional curcumin derivatives (PEG-CCM) + Amine-functionalized COFs (APTES-COF-1)	Doxorubicin	9.71 ± 0.13 wt%	-	[35]
4.	Porous covalent triazine framework PCTF	5,10,15,20-Tetraphenylporphyrin + Cyanuric chloride	IBU (ibuprofen)	19%	90% after 48 h	[36]
5.	t Cage-COF-TT (TT = triammonia–terephthalaldehyde),	Bis (tetraoxacalix (2) arene (2) triazine + Terephthalaldehyde	IBU (ibuprofen)	17.1 wt%	93% after 52 h	[37]
6.	t Cage-COF-TT (TT = triammonia–terephthalaldehyde),	Bis (tetraoxacalix (2) arene (2) triazine + terephthalaldehyde	FLU (fluorouracil)	21.4 wt%	93% after 52 h	[37]
7.	t Cage-COF-TT (TT = triammonia–terephthalaldehyde),	Bis (tetraoxacalix (2) arene (2) triazine + Terephthalaldehyde	CAP	22.3 wt%	94% after 52 h	[37]
8.	Porous aromatic frameworks PAF-6	Cyanuric chloride + Piperazine	IBU (ibuprofen)	0.35 gm	75% within 10 h	[38]
9.	Nanoscale covalent triazine polymer NCTP	Cyanuric chloride + Biphenyl	Doxorubicin	200 mg/g	80% at pH 4.8 over 48 h	[39]
10.	Porous covalent triazine framework PCTF-Mn	5,10,15,20-Tetraphenylporphyrin-Mn + Cyanuric chloride	IBU (ibuprofen)	23%	95% after 48 h	[40]
11.	Room temperature covalent organic framework-1 RT-COF-1	1,3,5-Tris(4-aminophenyl) benzene (TAPB) + 1,3,5-Benzenetricarbaldehyde (BTCA)	IBU	-	33% within 105 min	[41]
12.	Polyimide covalent organic framework-2 PI-2-COF	1,3,5-Triformylbenzene + 4,4′-Biphenyldiamine	5-FU	60 µg/mL	85% of initially loaded drug	[2,42]
13.	Polyimide covalent organic framework-3 PI-3-COF	1,3,5-Triformylbenze + 2,4,6-Tris (4-aminophenyl)-s-triazine	5-FU	32 µg/mL	85% of initially loaded drug	[2,42]
14.	Pemetrexed supramolecular framework (PMX@SOF)	Pyridinium-based tetracationic monomer (variable composition) + Cucurbit (8) uril ring	Pemetrexed	23	60 h	[43]
15.	1,3,5-triformylphloroglu 4-amino salicyl hydride (TpASH)	1,3,5-triformylphloroglu (Tp) + 4-Amino salicyl hydride (ASH)	5-FU Fluorouracil	12	72 h	[44]
16.	1,3,5-triformylphloroglu 4 amino benzo hydride (TpAPH)	1,3,5-triformylphloroglu (Tp) + 4-Amino benzo hydrides (APH)	5-FU Fluorouracil	12	72 h	[44]
17.	2,5-dihydroxyterephthalaldehyde 1,3,5-tris(4-aminophenyl) benzene COF (COF-DhaTab)	2,5-dihydroxyterephthalaldehyde+ 1,3,5-tris(4-aminophenyl) benzene	DOX Doxorubicin	35	Less than 7 days	[45]
18.	1,3,5-tris(4-aminophenyl) benzene -2,5-dimethoxyterephthaldehyde-COF (TAPB-DMTP-COF)	2,5-dimethoxyterephthaldehyde (DMTP) + 1,3,5-tris(4-aminophenyl) benzene (TAPB)	DOX Doxorubicin	-	-	[46]

## Data Availability

Not applicable.
